# Sequence changes in predicted promoter elements of *STK11/LKB1 *are unlikely to contribute to Peutz-Jeghers syndrome

**DOI:** 10.1186/1471-2164-6-38

**Published:** 2005-03-17

**Authors:** Nicholas CM Hearle, Ian Tomlinson, Wendy Lim, Victoria Murday, Edwin Swarbrick, Guan Lim, Robin Phillips, Peter Lee, John O'Donohue, Richard C Trembath, Patrick J Morrison, Andrew Norman, Rohan Taylor, Shirley Hodgson, Anneke Lucassen, Richard S Houlston

**Affiliations:** 1 Section of Cancer Genetics, Institute of Cancer Research, Sutton, UK; 2 Molecular and Population Genetics Laboratory, Imperial Cancer Research Fund, London, UK; 3 Section of Cancer Genetics, Institute of Cancer Research, Sutton, UK; 4 Dept of Medical Genetics, Yorkhill Hospital, Glasgow, UK; 5 Dept of Gastroenterology, New Cross Hospital, Wolverhampton, UK; 6 Dept of Gastroenterology, Epsom General Hospital, UK; 7 Polyposis Registry, St. Mark's Hospital, Watford Road, Harrow, UK; 8 Dept of Surgery, Hull Royal Infirmary, UK; 9 Dept of Gastroenterology, University Hospital Lewisham, London, UK; 10 Dept of Clinical Genetics, Leicester Royal Infirmary, Leicester, UK; 11 Department of medical genetics, Belfast City Hospital, UK; 12 Clinical Genetics Unit, Birmingham Women's Hospital, Birmingham, UK; 13 Molecular Genetics Unit, St Georges Hospital Medical School, London, UK; 14 Department of Clinical Genetics, St. Georges Hospital, London, UK; 15 Wessex Clinical Genetics Service, The Princess Anne Hospital, Southampton, UK; 16 Section of Cancer Genetics, Institute of Cancer Research, Sutton, UK

## Abstract

**Background:**

Germline mutations or large-scale deletions in the coding region and splice sites of *STK11/LKB1 *do not account for all cases of Peutz-Jeghers syndrome (PJS). It is conceivable that, on the basis of data from other diseases, inherited variation in promoter elements of *STK11/LKB1 *may cause PJS.

**Results:**

Phylogenetic foot printing and transcription factor binding site prediction of sequence 5' to the coding sequence of *STK11/LKB1 *was performed to identify non-coding sequences of DNA indicative of regulatory elements. A series of 33 PJS cases in whom no mutation in *STK11/LKB1 *could be identified were screened for sequence changes in the putative promoter defined by nucleotides -1090 to -1472. Two novel sequence changes were identified, but were found to be present in healthy individuals.

**Conclusion:**

These findings indicate that promoter sequence changes are unlikely to contribute to PJS.

## Background

Peutz-Jeghers syndrome (PJS; MIM 175200) is a rare autosomal dominant disorder typified by hamartomatous polyposis of the gastrointestinal tract and melanin pigmentation of the oro-facial region [1]. Although germline mutations in the coding sequence of the serine-threonine kinase gene *STK11/LKB1 *have been found to cause PJS [2,3], such mutations only account for up to 80% of cases [4-12]. In addition to locus heterogeneity [13] mutations in regulatory sequences of *STK11/LKB1 *may cause PJS.

Identifying regulatory genomic sequences through functional assays is time consuming and problematic. As natural selection is more likely to tolerate sequence changes in redundant, non-functional sequence than in functionally important sequences, regulatory elements in non-coding sequence will be highly conserved through evolution. Comparison of sequence between both closely related and highly divergent species therefore allows for the identification of non-coding sequence that has a high probability of being important to the regulation of gene transcription. This alternative approach to the identification of promoter elements is termed "phylogenetic foot printing" [14].

Here we describe the phylogenetic foot printing of the 5' upstream region of *STK11/LKB1 *and the sequence analysis of this region in a series of PJS cases in whom exonic and splice site mutations in the gene had been excluded.

## Results and discussion

**Figure 1:**
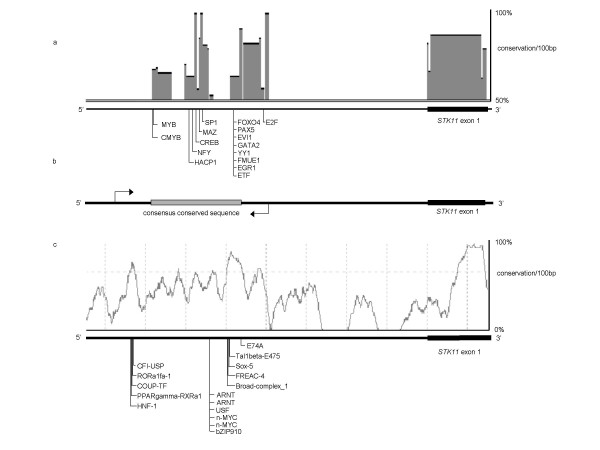
*in-silico *identification of the putative *STK11/LKB1 *promoter. (a) ECR browser output. Regions of high sequence conservation 5' of STK11 exon 1 are annotated with transcription factor binding sites as predicted by rVista v2.0 between nucleotides -981 to -1668. (c) ConSite output. Sequence of high conservation is annotated with predicted transcription factor binding sites (TFBS) as predicted by the JASPER TFBS database between nucleotides -1104 to -1613. (b) Ideogram showing consensus conserved region, defined at the 3' end by the E74A TFBS predicted ConSite, and at the 5' end by the CMYB TFBS predicted by rVista. Arrows indicate the genomic sequence analysed.

Figure 1 shows the evolutionary conserved region 5' of the coding sequence of *STK11 *as identified by the phylogenetic foot-printing programs ECR Browser [15] and CONSITE [16]. There was a high degree of concordance between the predictions from the programs. ECR Browser predicted three regions of high conservation upstream of *STK11*, encompassing nucleotides -981 to -1668 (nucleotide 0 representing the *STK11 *translation initiation signal). Sixteen transcription factor binding sites (TFBS) predicted by rVista [17] resided between nucleotides -1053 and -1472. ConSite predicted 27 TFBSs conserved between human and mouse between nucleotides -1090 and -1605. Through the integration of phylogenetic foot printing and TFBS prediction data from ECR browser/ rVista and ConSite, a consensus region containing predicted TFBS positions was identified between nucleotides -1090 to -1472.

Additional analysis using the TFBS prediction programs Transplorer (Biobase Biological Databases, Wolfenbüttel, Germany) and Proscan v1.7 [18] identified 9 binding sites between nucleotides -1142 and -1724, and 76 binding sites between nucleotides -881 and -1572 respectively, confirming the presence of regulatory elements within the consensus region. ECR Browser/ rVista and ConSite have previously been shown to correctly identify 88% and 66% of TFBS in functionally verified promoter elements respectively[15,16] and it is highly likely, therefore, that the consensus region encompassing nucleotides -1090 to -1472, contains *STK11/LKB1 *promoter elements.

DNA from a series of 33 PJS patients that did not harbour germline *STK11/LKB1 *mutations (mean age at diagnosis 14 years, range 1–38) was studied for mutations in the region between nucleotides -1001 to -1815, encompassing all TFBSs predicted by rVista and ConSite. Seven of the cases had a documented family history of PJS. The diagnosis of PJS in all cases was based on established criteria [1]. Three sequence changes were identified. The change G → C at position -1566 was found in four cases (three heterozygotes and one homozygote) and represented a previously documented single nucleotide polymorphism (rs3795061). An additional single nucleotide change at position -1268 (G → T) was identified in eight cases (seven heterozygotes and four homozygotes). A single sample of control DNA used as a sequence reference was also found to be homozygous for the change. Finally, a two base pair deletion coupled with a single base pair insertion at position -1709 (n-1709insTdelCC) was identified in one case. A series of healthy population controls (n = 92) was screened by High Performance Liquid Chromatography (HPLC) and one individual (1/92) was found to harbour the sequence change. None of these three sequence changes identified were therefore deemed to be potentially pathogenic.

There is a high degree of redundancy in promoter elements of genes, however point mutations in promoter regions of PTEN and MLH1 have been reported to be disease causing [19,20]. To investigate the possibility of large-scale deletions or insertions undetectable by straightforward PCR primers P1Fwd and P3Rev were used to amplify an 814 bp fragment with products visualised on a 2% agarose gel. No large-scale deletions or insertions were detected in any of the patients.

## Conclusion

As understanding of the contribution of coding sequence changes to disease becomes clearer, attention will focus on regulatory elements of genes. Phylogenetic foot printing using programs such as ECR browser and ConSite present potentially powerful tools in identifying regulatory elements, enabling the analysis of these sequences without time consuming functional studies. Although the efficiency of in-silico delineation of promoter elements has not been rigorously evaluated, ECR Browser/ rVista and ConSite have been shown to correctly identify 88% and 66% of TFBS respectively[15,16]. On the basis of our findings, however, it appears unlikely (upper 95% confidence interval, prevalence; 9%) that mutations in the promoter region of *STK11/LKB1 *are responsible for PJS cases not attributable to exonic sequence changes.

## Methods

### Bioinformatics

Phylogenetic foot printing of 3 Kb of sequence upstream of human and mouse *STK11/LKB1 *(NT_011255) was performed using the promoter predication programs ECR Browser [15] and CONSITE [16]. For both programs a conservation cut-off of 70% identity over 100 base pairs was adopted, in accordance with published criteria [21,22]. Regions identified by ECR Browser with greater than 70% conservation were analysed using the TFBS prediction program rVista v2.0 [17]. Conserved regions identified by ConSite were annotated with TFBSs based on the JASPAR database [23] and links provide sequence information. Further analysis was carried out using the TFBS prediction programs Proscan version1.7 [18] and Transplorer (Biobase Biological Databases, Wolfenbüttel, Germany). The promoters and TFBS predicted by each program were aligned to delineate a consensus region indicative of a putative promoter.

### Mutational analysis

The possession of germline mutations in *STK11/LKB1*, including a large-scale deletion of the gene, was excluded using methods previously described [24]. Mutational analysis of the minimal consensus conserved region was carried out by direct DNA sequencing in both directions using the BigDye v3.1 Terminator Cycle Sequencing Ready Reaction Kit in conjunction with an ABI3100 semi-automated genetic analyser (Applied Biosystems, Foster City, USA). Overlapping oligonucleotides amplifying three fragments spanning the region were designed using the Primer3 program [25]: P1Fwd – GCACAGGAGGGTTCAATATTTTC, P1Rev – TTGCGGACCTGGAAGGAG, P2Fwd – ACTGGAATTGGCCACTTTGT, P2Rev – GATACAGCGCGCTCATTG, P3Fwd – GTCTCCCCATGCCTGCTTC, P3Rev – GGCCCAGCCCATCCAAGG. Predicted PCR products were subjected to searches of the genome using the BLAST program [26] to confirm specificity. Chromatograms were analysed by two independent researchers using the programs Chromas [27] and MutationSurveyor (SoftGenetics, State College, USA). High performance liquid chromatography was performed using the WAVE system (Transgenomics, Omaha, USA).

## Authors' contributions

NCMH performed all bioinformatical and molecular studies and drafted the manuscript. IT, WL, VM, ES, GL, RP, PL, JO, RT, PJM, AN, RCT, SH and AL contributed cases of PJS to the study. RSH conceived the study and drafted the manuscript, and participated in the study's design and coordination. All authors read and approved the final manuscript.
